# Real-Time Forecasting of Subsurface Inclusion Defects for Continuous Casting Slabs: A Data-Driven Comparative Study

**DOI:** 10.3390/s23125415

**Published:** 2023-06-07

**Authors:** Chihang Wei, Zhihuan Song

**Affiliations:** 1 School of Information Science and Technology, Hangzhou Normal University, Hangzhou 311121, China; 2 State Key Laboratory of Industrial Control Technology, Zhejiang University, Hangzhou 310027, China

**Keywords:** real-time forecasting, subsurface inclusion defects, data-driven methods, discriminant analysis, stack autoencoder

## Abstract

Subsurface inclusions are one of the most common defects that affect the inner quality of continuous casting slabs. This increases the defects in the final products and increases the complexity of the hot charge rolling process and may even cause breakout accidents. The defects are, however, hard to detect online by traditional mechanism-model-based and physics-based methods. In the present paper, a comparative study is carried out based on data-driven methods, which are only sporadically discussed in the literature. As a further contribution, a scatter-regularized kernel discriminative least squares (SR-KDLS) model and a stacked defect-related autoencoder back propagation neural network (SDAE-BPNN) model are developed to improve the forecasting performance. The scatter-regularized kernel discriminative least squares is designed as a coherent framework to directly provide forecasting information instead of low-dimensional embeddings. The stacked defect-related autoencoder back propagation neural network extracts deep defect-related features layer by layer for a higher feasibility and accuracy. The feasibility and efficiency of the data-driven methods are demonstrated through case studies based on a real-life continuous casting process, where the imbalance degree drastically vary in different categories, showing that the defects are timely (within 0.01 ms) and accurately forecasted. Moreover, experiments illustrate the merits of the developed scatter-regularized kernel discriminative least squares and stacked defect-related autoencoder back propagation neural network methods regarding the computational burden; the F1 scores of the developed methods are clearly higher than common methods.

## 1. Introduction

Continuous casting slabs are a kind of indispensable foundation material in economic construction, the quality of which crucially guarantees the safety and quality of the final products [1,2]. A subsurface inclusion is one of the most frequent defects that affects the inner quality of the continuous casting slabs. Specifically, subsurface inclusion defects refer to irregular and discontinuous slag chunks embedded in the surface or 2∼10 mm under the surface. It can cause serious defects in the resultant hot rolling or cold rolling products, increasing the defective index, the probability of breakout accidents, and the complexity of the hot rolling process [3,4,5].

Subsurface inclusion defects are a critical problem in the steel industry. Technologies to solve this problem have been extensively studied in the past by both academia and industry, and can be classified into mechanism-model-based methods, physical-parameter-based methods, and process knowledge (metallurgy function)-based methods. (1) Mechanism-model-based methods firstly involve the construction of a mechanism model of the continuous casting process. Then analytical solutions are obtained by numerical simulation tools and subsurface inclusion defects are predicted by the constructed mechanism model [6,7]. The main disadvantages are that it is usually hard to obtain an accurate mechanism model and the efficiency of practical application is greatly reduced by artificial assumptions. (2) Physical-parameter-based methods utilize mechanical and electrical technologies, optical technology, and the pickling test for hot-rolled steel to detect slab surfaces, including eddy current testing, the induction heating method, optical detection, photoelectric integration, macrofractography, etc. The disadvantages include that they are highly time consuming, inefficient, and highly expensive, requiring a complete set of related equipment. (3) Process knowledge (metallurgy function)-based methods utilize the metallurgy function, constructed to describe the functional relationship between quality defects of slabs and their related technological parameters, to forecast the quality defects and their orders of severity [8]. The disadvantages are obvious: the insolubility of complex nonlinear features, the difficulty of setting the weights of all technological parameters, and the inability to describe the causes of defects.

With the great improvements in measurement techniques and information technology, a large amount of process data can be expediently collected [9,10,11,12]. Data-driven methods have attracted increasingly more attention, and are characterized by simple implementation, a broad applicability, and fewer requirements for a model mechanism or process knowledge [13,14,15,16,17,18]. The advantages over other techniques are highlighted in the following.

(1) Subsurface inclusion defects cannot be detected by traditional detectors until the slabs have been rolled in the subsequent hot rolling or cold rolling processes, since the surface temperature is quite high and the defects may be buried deep inside. Thus, there exists a large time delay between defect generation and defect detection. The machines may operate in poor conditions for a long time and the use of defective slabs may waste valuable time and resources. With the help of data-driven modeling and prediction methods, the defects can be predicted based on process variables, which can be collected in real-time during the manufacturing of continuous casting slabs. “Real-time” signifies that the forecasting information is obtained immediately following manufacturing of the slab (even if it is quite hot), based on measurements of process variables such as temperature, flow rate, and speed. Moreover, real-time forecasting is nondestructive, while some detection devices make destructive incisions to monitor defects. It is noted that data-driven forecasting models are not incompatible with traditional devices, but they can make up for each other’s shortcomings. Data-driven models can provide defect prediction using the process variables with hardly any delay, while traditional detectors can help to model, revise, and update the data-driven models.
(2) Data-driven methods do not need precise mechanistic information or expert knowledge, and are characterized by precision, simplicity, straightforwardness, economic value, and universality without requiring strong first-principle knowledge. Process data have become abundant with the extensive use of distributed control systems (DCSs), which reflect the dependency relationship between the quality defects and their origin. Furthermore, with the proposal of “Industry 4.0”, steel enterprises have updated their data collection and storage systems, making it easy to acquire rich data, ranging from Level 1: basic automation data to Level 4: enterprise resource plan data. This lays the foundation for data-driven methods.
(3) With timely and accurate defect information, slabs with defects would thus not be used in the subsequent production processes, reducing the defective index and improving the production efficiency. Furthermore, real-time defect information contributes to control system performance evaluation and real-time feedback control, enabling adjustment of the control strategy to eliminate defects. It can also help operators take appropriate action to prevent further deterioration of the operating conditions.

In the present paper, a comparative study is carried out on data-driven defect forecasting methods. There are many kinds of data-driven models in the literature; several basic but representative methods, including LDA [19], LR [20], SVM [21,22], and XGBoost [23], are introduced to provide a preliminary verification and demonstration of the feasibility and efficiency of data-driven methods. For SVM, the radial basis function (Gaussian) kernel is used, from which the parameters can be determined. This paper also introduces an intuitionistic method based on practical evaluation metrics to determine the parameters for XGBoost, including the maximum depth, minimum sum of instance weight, maximum delta step, subsample, and learning rate. Many data mining techniques can be utilized in defect forecasting, such as clustering, random forest, regression analyses, association rules, and anomaly (extreme value) analyses. Clustering is an unsupervised machine learning technique that automatically discovers natural grouping in data without coming up with a specific hypothesis; it is not quite suitable for the topic as it is incapable of extrapolation. For example, Lukauskas et al. proposed an extension to the clustering method based on the modified inversion formula density estimation to resolve previous method limitations regarding outliers [24]. Random forest is an ensemble learning method for classification, regression, and others that constructs a multitude of decision trees [25]. The previously mentioned XGBoost is more suitable for complex datasets compared to random forest. Regression analyses, similar to the previously mentioned LR, are a technique for estimating the relationships between a dependent variable and one or more independent variables [26]. For example, the primary objective function arising from probability regularization was extended in support vector regression, leading to an automatic selection of hyperparameters. This new algorithm was tested on public benchmark datasets, such as the QSAR aquatic toxicity dataset (qsar), the yacht hydrodynamics dataset (yachts), and the concrete compressive strength dataset (concrete) [27]. Anomaly detection can identify items which deviate significantly from the majority of the data and do not conform to a well-defined notion of normal behavior [28,29]. This method can work with significantly imbalanced data (positive samples are rare). However, the advantage of this technique is no longer clear when the imbalance problem vanishes.

Traditional data-driven forecasting methods mainly focus on discovering the low-dimensional embeddings within a certain class separability, and the forecasting result can be calculated by supplementary classifiers based on these embeddings. There exist two main procedures: one extracts features under some criterion and the other builds a classifier based on these features. In order to design a more suitable model and practically improve the prediction of subsurface inclusion defects, a novel model, named SR-KDLS, is developed in this paper to directly provide forecasting information instead of low-dimensional embeddings. The objective is designed to directly focus on the forecasting performance by penalizing the difference between the real class information and the predict class information through least squares. To further improve the forecasting accuracy, discriminative data information is integrated into the regularization term to pursue both intra-class compactness and inter-class separability. Specifically, the between-class scatter is maximized while the within-class scatter is simultaneously minimized to group samples from the same class and synonymously separate samples from different classes in the feature space to improve the classification performance. The novel model is used with the reproducing kernel Hilbert space (RKHS) setting as a scatter-regularized optimization, guaranteeing both flexibility and feasibility.

Deep learning has become increasingly popular in the field of process system engineering; classical applications include fault detection, fault traceability, virtual sensors, etc. [30,31,32,33,34,35,36,37]. For example, Zhang et al. developed a conditional variational generative adversarial network (CVAE-GAN) model for multiclass wind turbine bearing fault diagnoses by merging the variational autoencoder (VAE) with the deep learning generative adversarial network (GAN) [33]. Guo et al. proposed a deep multiple attention soft sensor (DMASS) model with multiple attention mechanisms and a deep learning framework, ensuring the self-interpretability of data selection and sensor modeling, and tried to integrate these originally independent phases into a single scheme [34]. Zhang et al. proposed a three-layer fusion fault diagnosis method based on deep learning to deal with multifault diagnoses in complex industrial processes [35]. The naive stack autoencoder (SAE), constructed by hierarchically stacking autoencoders (AEs), is one of the most widely adopted deep learning techniques, due to its strong ability to extract informative features from the original data. These extracted features improve the performance of traditional predictors or classifiers. For example, the novel quality-driven regularization (QR) is proposed for deep network SAEs to learn quality-related features from industrial process data, changing the loss function to control the weights of the different input variables [36]. To improve the performance of early fault detection in large-scale nonlinear industrial processes, the decentralized adaptively weighted stacked autoencoder (DAWSAE)-based fault detection method was proposed with local and global adaptively weighted feature vectors and residual vectors [37]. SAEs work well in unsupervised learning tasks such as fault detection; however, they cannot guarantee extraction of defect-related features for the promotion of real-time forecasting of subsurface inclusion defects. This is because the pretraining technique of the AEs in each layer is unsupervised (self-reconstruction), resulting in it learning the features that largely represent the input data, but not those related to defect information. In this paper, the stacked defect-related autoencoder (SDAE) and hierarchically stacked defect-related autoencoder (DAE) are developed to predict defects. In each DAE, the inputs are low-layer features, while the outputs are the reconstructed low-layer features combined with reconstructed defect-related information. During layer-by-layer pretraining, supervised self-reconstruction is adopted to extract defect-related features that can largely improve the forecasting performance. Successively, all the DAEs are hierarchically stacked to learn increasingly deeper defect-related features. Fine tuning of the whole network is finally performed. To predict real-time defects, a back propagation neural network (BPNN) is trained based on the deepest features learned by the SDAEs. The BPNN also helps to fine tune the parameters of the SDAE to further improve the performance.

In the strictest sense, this paper solves classification tasks that make predictions based on data-driven methods upon measurements of process variables. Traditional detection devices detect defects with a large time delay after slabs have been rolled by hot rolling or cold rolling processes; on the contrary, the data-driven methods in this paper can detect defects with hardly any delay, seemingly playing the role of a “forecaster” to some extent. Thus, this paper title presents “forecasting” with traditional detecting methods. All of the above techniques will be further discussed in the remainder of this paper. In Section 2, the backgrounds of the continuous casting process and evaluation metrics are provided. Section 3 and Section 4 present the development of the SR-KDLS model and SDAE model, respectively, with detailed derivations. Subsequently, in Section 5, application case studies are evaluated and a comparison is performed of different data-driven defect forecasting methods based on a real-life slab casting process with eight categories of casting slabs. Finally, some conclusions and outlooks are presented in the final section.

## 2. Preliminaries

In this section, the continuous casting process is introduced. As is common knowledge, the background knowledge of the four representative data-driven forecasting methods (LDA, LR, SVM, and XGBoost) is omitted for briefness. To facilitate an easy understanding, the background of evaluation metrics for forecasting performance is reviewed.

### 2.1. The Continuous Casting Process

**Figure 1:**
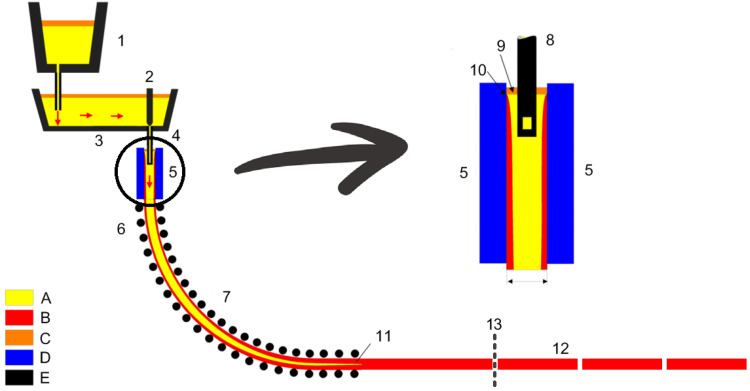
Sketch map of the continuous casting process. Legend: A: Liquid metal; B: Solidified metal; C: Slag; D: Water-cooled copper plates; E: Refractory material; 1: Ladle; 2: Stopper; 3: Tundish; 4: Shroud; 5: Mold; 6: Roll support; 7: Turning zone; 8: Shroud; 9: Bath level; 10: Meniscus; 11: Withdrawal unit; 12: Slab; 13: Torch cutting point.

The continuous casting process is one of the most important procedures in steel manufacturing. It produces continuous casting slabs. In this process, molten steel is first transported to crystallizers through steel tundishes and ladles, where casting powder is added to preserve heat, prevent secondary oxidation of the molten steel, and absorb impurities. In the crystallizers, the molten steel cools and solidifies to soft billet covered by a protective shell with a certain thickness, which is then drawn and straightened by straightening machines and dummy devices. Finally, it is cut into slabs by torch cutting machines. Figure 1 presents a sketch of the continuous casting process, modified from the original version (https://en.wikipedia.org/wiki/Continuous_casting (accessed on 1 June 2023)), where a torch cutting point has been added. The continuous casting slab is a raw material for subsequent processes such as hot rolling or cold rolling, where the final products, including cold-rolled steel or coils, hot-rolled steel or coils, and bar steel, are manufactured.

### 2.2. Evaluation Metrics

**Figure 2:**
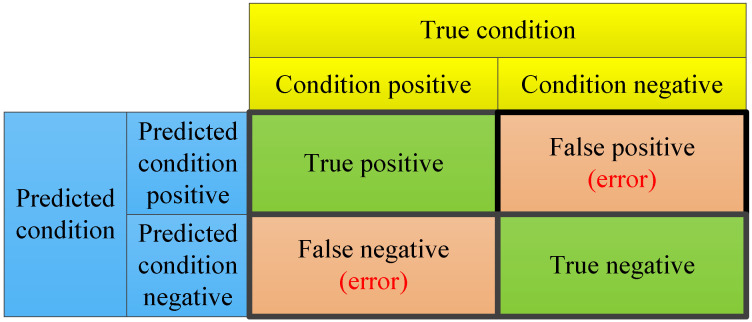
Relations among true positive, false positive, true negative, false negative, true condition, and predicted condition.

The slabs made by the continuous casting process are either normal (containing defects) or defective (containing subsurface inclusion defects). In general, a normal slab is labeled as “negative”, while a defective slab is labeled as “positive”. The forecasting model predicts a slab as either being negative or positive. There are four cases of the result after an instance being forecasted: true positive, false positive, true negative, and false negative. Figure 2 illustrates their relations. For a convenient comparison, three general metrics are utilized:

(1) $$\begin{array}{cc} \; & Precision=Truepositive/(Truepositive+Falsepositive)\\ \; & Recall=Truepositive/(Truepositive+FalsePrecision)\\ \; & F1=2\times Precision\times Recall/(Precision+Recall) \end{array}$$

where precision (also called the positive predictive value) is the fraction of relevant instances among the retrieved instances; recall (also known as the sensitivity) is the fraction of the total amount of relevant instances that was actually retrieved; and F1 (also called the F-measure) is the harmonic mean of precision and recall.

As false negatives are undesirable in subsurface inclusion defect forecasting, F1 is chosen in this paper to simultaneously pursue a high precision and a high recall. In practice, both false positives and false negatives are unwanted error situations. A high precision and a high recall are desirable, resulting in a high F1. Unfortunately, precision and recall are often at odds for a given forecasting model with the same data. That is, improving the precision typically reduces the recall and vice versa.

## 3. Scatter Regularized Kernel Discriminative Least Squares

Data-driven methods have been widely used in forecasting tasks. Despite their favorable properties, the forecasting performance could be further improved. In order to design a more suitable model, this paper develops the SR-KDLS model to improve the real-time forecasting performance for subsurface inclusion defects. It would directly provide forecasting information, instead of low-dimensional embedding, and is used with the RKHS setting with one compact optimization step. Specifically, the objective function is combined with a loss function and a regularization term. The least squares loss function is designed to directly focus on the forecasting performance by penalizing large differences between the real class information and the predicted class information. Furthermore, the regularization term exploits the between-class scatter and within-class scatter of the data to pursue both intra-class compactness and inter-class separability.

For a Mercer kernel κ:x×x→R, there is an associated RKHS $H_{\kappa }$ of functions x→R with the corresponding norm ${\left||\;|\right|}_{\kappa }$ [38]. Specifically, the optimization of SR-KDLS is expressed as,

(2) $$\begin{array}{c} f^{*}=arg\min_{f\in H_{\kappa }}V\left(y,f\left(x\right)\right)+\gamma R^{Scatter} \end{array}$$

where V(y,f(x)) is the loss function to penalize large differences between the real class information *y* and the predicted class information f(x). *f* is the prediction function. Note that the defective information *y* is either 0 (negative) or 1 (positive). In this paper, $V\left(y,f\left(x\right)\right)=\sum_{i=1}^{N}{\left(y_{i}-f\left(x_{i}\right)\right)}^{2}$ is simply chosen as the least squares loss function. $R^{Scatter}$ is the scatter-regularization term, derived from discriminative information. γ is the parameter that balances the order of magnitudes between V(y,f(x)) and $R^{Scatter}$. The classical Representer Theorem states that the solution to this minimization problem (2) exists with respect to *f* in $H_{\kappa }$ [38] and can be written as

(3) $$f^{*}\left(x\right)=\sum_{i=1}^{N}\alpha_{i}\kappa \left(x_{i},x\right)$$

Therefore, the problem in (2) is greatly reduced to optimization coefficients $\alpha_{i}$ over the finite dimensional space. To this end, both the loss function $V(y_{i},f\left(x_{i}\right))$ and regularization $R^{Scatter}$ should be formulated in terms of $\alpha_{i}$ and κ(·,·). For notation simplicity and derivation ease, the inner product matrix (Gram matrix) is expressed as $K\in R^{N\times N}$ and the ij−th element of K is defined as $K_{ij}=\kappa \left(x_{i},x_{j}\right)=\Phi \left(x_{i}\right)\cdot \Phi \left(x_{j}\right)=\Phi {\left(x_{i}\right)}^{⊺}\Phi \left(x_{j}\right)$, where κ(·,·) is the kernel function and Φ(·) is an implicit kernel. In addition, $\alpha ={\left[\alpha_{1}\cdots \alpha_{N}\right]}^{⊺}$ and $y={\left[y_{1}\cdots y_{N}\right]}^{⊺}$, where the element $y_{n}$ is binary.

The construction and derivation of $V(y_{i},f\left(x_{i}\right))$, $R^{Scatter}$ and the compact optimization are explicitly presented in the following.

### 3.1. Construction of the Loss Function

The loss function can be easily reformulated as

(4) $$V\left(y,f\left(x\right)\right)=\sum_{i=1}^{N}{\left(y_{i}-f\left(x_{i}\right)\right)}^{2}={\left(y-K\alpha \right)}^{⊺}\left(y-K\alpha \right)$$

### 3.2. Construction of the Regularization Term

In the SR-KDLS model, the between-class scatter is to be maximized to separate samples from different classes, while the within-class scatter is to be minimized to group samples from the same class. The $R^{Scatter}$ is constructed by integrating the two scatters together. To accomplish this, various indices that quantify the within-class scatter and the between-class scatter in the unfolded feature space should be defined.

#### 3.2.1. Within-Class Scatter

The within-class scatter, $\sigma_{k}^{W}$, for a specific class *k* and the overall within-class scatter, $\sigma^{W}$, can be calculated by,

(5) $$\begin{array}{cc} \; & \sigma_{k}^{W}=\sum_{x_{i}\in G_{k}}{∥f\left(x_{i}\right)-f\left({\bar{x}}_{k}\right)∥}^{2}/N_{k}\in R^{1}\\ \; & \sigma^{W}=\sum_{k=1}^{K}\sigma_{k}^{W} \end{array}$$

respectively.

Similar to LDA, ${\bar{x}}_{k}=\sum_{x_{i}\in G_{k}}x_{i}/N_{k}$ denotes the central point (mean) of class *k* and represents the class location. However, Equation (5) does not work since it can not be formulated in terms of α and K. It is noted that K only includes the training data samples, but the central point ${\bar{x}}_{k}$ for each class usually does not belong to the training data. Therefore, the measurement of within-class scatter is slightly modified; one “representative sample” $x_{s^{k}}$ is selected as the nearest sample to ${\bar{x}}_{k}$ in $G_{k}$ for each class to replace the center point ${\bar{x}}_{k}$ in (5), where the subscript $s^{k}$ is its serial number in ${\left\{(x_{i},y_{i})\right\}}_{i=1}^{N}$,

(6) $$x_{s}^{k}=\arg\min_{x_{i}\in G_{k}}\left|\right|x_{i}-{\bar{x}}_{k}{\left|\right|}^{2}$$

Then,

(7) $$\begin{array}{cc} \; & \sigma_{k}^{W}\\ \; & =\;\sum_{x_{i}\in G_{k}}f{\left(x_{i}\right)}^{⊺}f\left(x_{i}\right)-2f{\left(x_{i}\right)}^{⊺}f\left(x_{s^{k}}\right)+f{\left(x_{s^{k}}\right)}^{⊺}f\left(x_{s^{k}}\right)/N_{k}\\ \; & =\;\sum_{x_{i}\in G_{k}}\;{\left[K_{i\cdot }\alpha \right]}^{⊺}K_{i\cdot }\alpha \;-\;2{\left[K_{i\cdot }\alpha \right]}^{⊺}K_{s^{k}\cdot }\alpha \;+\;{\left[K_{s^{k}\cdot }\alpha \right]}^{⊺}K_{s^{k}\cdot }\alpha /N_{k}\\ \; & =\alpha^{⊺}\left[\sum_{x_{i}\in G_{k}}K_{i\cdot }^{⊺}K_{i\cdot }-2K_{i\cdot }^{⊺}K_{s^{k}\cdot }+K_{s^{k}\cdot }^{⊺}K_{s^{k}\cdot }/N_{k}\right]\alpha \\ \; & =\alpha^{⊺}V_{k}^{W}\alpha \end{array}$$

$\sigma^{W}=\alpha^{⊺}V^{W}\alpha$ can be sequentially reformulated, where $V^{W}=\sum_{k=1}^{K}V_{k}^{W}$.

#### 3.2.2. Between-Class Scatter

For the same reason, i.e., to replace ${\bar{x}}_{k}$ with $x_{s^{k}}$ in $\sigma_{k}^{W}$ to express the objective function in terms of the elements of K, the measurement of between-class scatter in the projected feature space is innovatively calculated as

(8) $$\begin{array}{cc} \; & \sigma^{B}\\ \; & =\sum_{k_{i},k_{j}=1}^{K}{∥f\left(x_{s^{k_{i}}}\right)-f\left(x_{s^{k_{j}}}\right)∥}^{2}\\ \; & =\alpha^{⊺}\;\;\left[\sum_{k_{i},k_{j}}\left(K_{s^{k_{i}}.}^{⊺}K_{s^{k_{i}}.}\;\;-\;\;2K_{s^{k_{i}}.}^{⊺}K_{s^{k_{j}}.}\;\;+\;K_{s^{k_{j}}.}^{⊺}K_{s^{k_{j}}.}\right)\right]\;\alpha \\ \; & =\alpha^{⊺}V^{B}\alpha \end{array}$$

#### 3.2.3. The Regularization Term

On the basis of $\sigma^{W}$ and $\sigma^{B}$, $R^{Scatter}$ is constructed as

(9) $$R^{Scatter}=\sigma^{W}-\frac{\sigma^{B}}{K}=\alpha^{⊺}V\alpha$$

where $V=V^{W}-\frac{V^{B}}{K}$. The denominator *K* is present to balance the order of magnitudes between $\sigma^{W}$ and $\sigma^{B}$.

### 3.3. Optimization

Substituting the Representer Theorem (3), reformulated loss function (4), and the scatter-regularization item (9) into the compact optimization (2), the convex differentiable objective function with respect to α is given as,

(10) $$\alpha^{*}=arg\min{\left(y-K\alpha \right)}^{⊺}\left(y-K\alpha \right)+\gamma \alpha^{⊺}V\alpha$$

The derivative of the objective function vanishes at the minimizer. Let the derivative of (10) with respect to α approach zero,

(11) $${\left(y-K\alpha \right)}^{⊺}\left(-K\right)+\gamma \alpha^{⊺}V=0$$

which leads to the following solution:

(12) $$\alpha^{*}={\left[KK+\gamma V\right]}^{-1}Ky$$

To make a prediction $y_{new}=f\left(x_{new}\right)$ at a query sample $x_{new}$, the forecasting system can be successively constructed

(13) $$y_{new}=f\left(x_{new}\right)=\left[\kappa \left(x_{1},x_{new}\right),\cdots ,\kappa \left(x_{N},x_{new}\right)\right]\alpha^{*}=K_{new\cdot }\alpha^{*}$$

where

(14) $$K_{new\cdot }=\left[\kappa \left(x_{1},x_{new}\right),\cdots ,\kappa \left(x_{N},x_{new}\right)\right]$$

### 3.4. SR-KDLS-Based Forecasting

**Figure 3:**
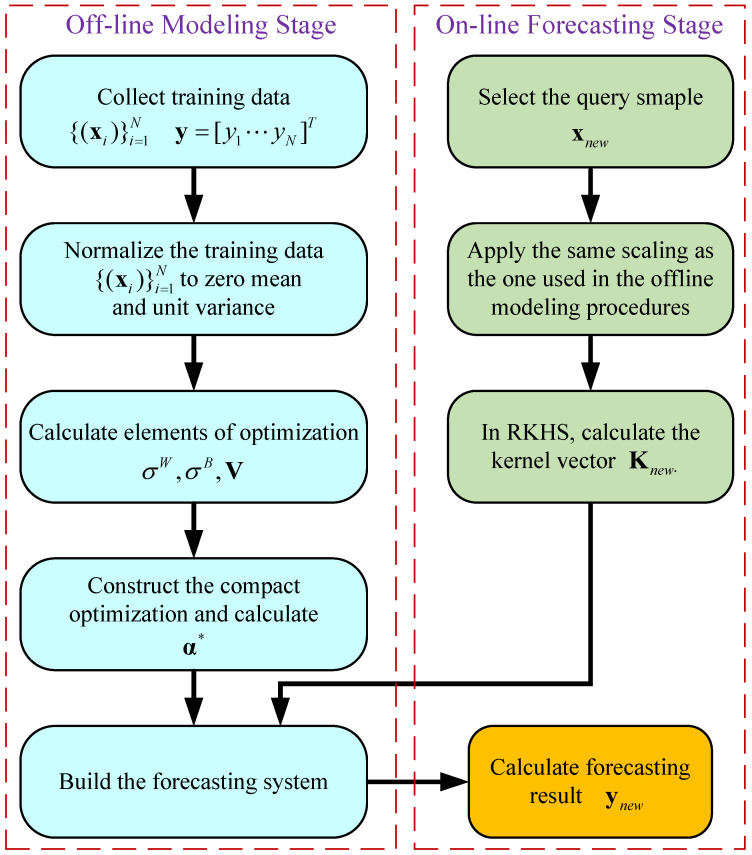
Flowchart of SR-KDLS-based defects forecasting.

The detailed procedures of the offline modeling stage and online forecasting stage of the proposed SR-KDLS-based forecasting method are listed in Algorithms 1 and 2, respectively. Note that only training data x should be normalized before modeling. To summarize, Figure 3 illustrates a flowchart of SR-KDLS-based forecasting.
**Algorithm 1** Off-line Modeling Stage of SR-KDLS.1:Collect training data ${\left\{(x_{i})\right\}}_{i=1}^{N}$ and $y={\left[y_{1}\cdots y_{N}\right]}^{⊺}$;2:Normalize ${\left\{(x_{i})\right\}}_{i=1}^{N}$ to zero mean and unit variance;3:Calculate $x_{s^{t}}$ for each class *t* using (6);4:Calculate the within-class scatter $\sigma_{k}^{W}$ for each class *t* using (7);5:Calculate the total within-class scatter $\sigma^{W}$;6:Calculate the between-class scatter $\sigma^{B}$ using (8);7:Construct the regularization term $R^{Scatter}$ and V (9);8:Calculate $\alpha^{*}$ using (12);9:Construct the forecasting system using (13).

**Algorithm 2** On-line Forecasting Stage of SR-KDLS.
1:Select the query sample $x_{new}$;2:Apply the same scaling as the one used in the offline modeling stage;3:Calculate the kernel vector using (14);4:Calculate the corresponding forecasting result using (13).


It is noted that SR-KDLS is currently designed for binary forecasting in this paper, but it can easily be extended to a more generalized form for multi-class forecasting as the scatters are already in a multi-class form. The label information $y_{i}$ should be encoded as one-hot and the least square loss function should be reformulated as $V\left(y,f\left(x\right)\right)=\sum_{i=1}^{N}{∥y_{i}-f\left(x_{i}\right)∥}^{2}$. Furthermore, note that other loss functions can be chosen, such as the hinge loss function, and other regularization terms can be integrated into the framework of scatter-regularized function learning, which would extend the scope of future studies and applications.

## 4. Stacked Defect-Related Autoencoder

The classical SAE has a strong ability to extract informative features from the original data in a layer-by-layer manner. However, it cannot guarantee the extraction of defect-related features for promoting real-time forecasting of subsurface inclusion defects. This is because feature learning is executed to largely represent the input data, and not the related defect information. In this paper, a defect-related autoencoder and a stacked defect-related autoencoder are successively developed.

### 4.1. Defect-Related Autoencoder

A simple AE simply reconstructs the input data and provides extracted features in the hidden layer. It ignores defect information; thus, it cannot extract defect-related features for promoting real-time forecasting of subsurface inclusion defects. Too much defect-unrelated information would occupy the information space of the extracted features. Furthermore, subsequent levels would then learn deeper features of these defect-unrelated features, and defect-related information would become increasingly rare. For suitability in actual applications, a defect-related autoencoder (DAE) is designed by introducing defect-related information in the training procedure.

**Figure 4:**
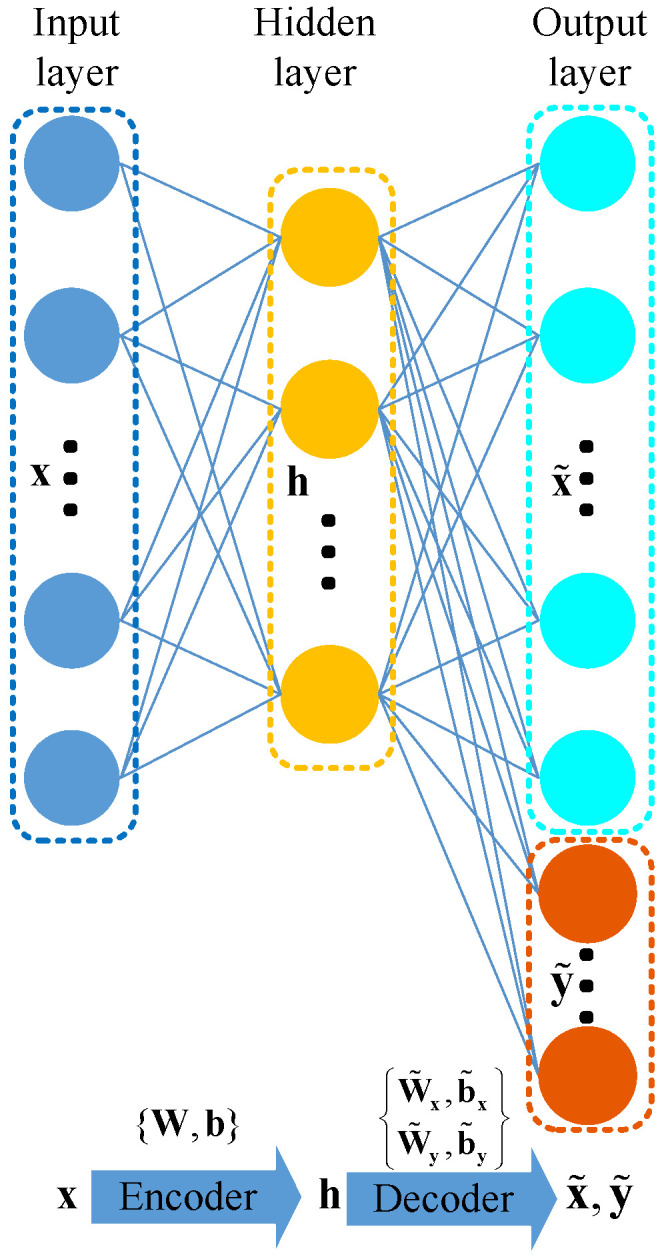
Network structure of the DAE.

Specifically, the DAE consists of three layers (the input, hidden, and output layers). While the former two layers remain the same as a simple AE, the output layer combines the reconstructed input variables and reconstructed defect-related information. Figure 4 illustrates the network structure of a DAE, where the blue dots, yellow dots, cyan dots, and red dots represent the input variables (denoted as $x\in R^{d_{x}}$), the hidden variables (denoted as $h\in R^{d_{h}}$), the reconstructed input variables (denoted as $\tilde{x}\in R^{d_{x}}$), and the reconstructed class information variables (denoted as $\tilde{y}\in R^{d_{y}}$), respectively. The symbols $d_{x}$, $d_{h}$, and $d_{y}$ denote the dimensions of the input variables, the hidden variables, and the class information variables, respectively. The symbols {W,b} denote the connecting parameters from the input layer to the hidden layer in the encoder. The symbols $\left\{{\tilde{W}}_{x},{\tilde{b}}_{x}\right\}$ denote one part of the connecting parameters in the decoder from the hidden layer to the reconstructed input variables $\tilde{x}$ in the output layer, while $\left\{{\tilde{W}}_{y},{\tilde{b}}_{y}\right\}$ denote the other part of the connecting parameters in the decoder from the hidden layer to the reconstructed class information variables $\tilde{y}$ in the output layer.

In the encoding procedure of the DAE, input variables $x=\left[x^{\left(1\right)},\cdots ,x^{\left(d_{x}\right)}\right]\in R^{d_{x}}$ are encoded to hidden variables $h=\left[h^{\left(1\right)},\cdots ,h^{\left(d_{h}\right)}\right]\in R^{d_{h}}$ with the following mapping relation

(15) h=f(Wx+b)

where *f* is an element-wise nonlinear activation function. W and b are the encoder weight matrix and bias vector, respectively. Then, in the decoding procedure of the DAE, hidden variables h are decoded to the reconstructed input variables $\tilde{x}$ and the reconstructed class information variables $\tilde{y}$ with the following mapping relations

(16) $$\begin{array}{cc} \tilde{x}= & g({\tilde{W}}_{x}h+{\tilde{b}}_{x})\\ \tilde{y}= & g({\tilde{W}}_{y}h+{\tilde{b}}_{y}) \end{array}$$

where ${\tilde{W}}_{x}$ and ${\tilde{b}}_{x}$ are the decoder weight matrix and bias vector for $\tilde{x}$, respectively, and ${\tilde{W}}_{y}$ and ${\tilde{b}}_{y}$ are the decoder weight matrix and bias vector for $\tilde{y}$, respectively. Note that the activation function from the hidden layer to the reconstruction layer is the same for both $\tilde{x}$ and $\tilde{y}$.

The encoder activation function, *f*, and the decoder activation function, *g*, are usually nonlinear functions such as the sigmoid function, the tanh function, or the rectified linear unit function in order to capture nonlinear relationships. Given the training data ${\left\{\left(x_{i},y_{i}\right)\right\}}_{i=1}^{N}$, where *N* denotes the number of training samples, the parameters set for the DAE $\theta =\{W,b,{\tilde{W}}_{x},{\tilde{b}}_{x},{\tilde{W}}_{y},{\tilde{b}}_{y}\}$ can be obtained by minimizing the following reconstruction loss function in the mean squared error form,

(17) $$J_{DAE}\left(\theta^{2}\right)=\frac{1}{2N}\sum_{i=1}^{N}\left({∥x_{i}-{\tilde{x}}_{i}∥}^{2}+\lambda {∥y_{i}-{\tilde{y}}_{i}∥}^{2}\right)$$

where λ is a supplement to balance the order of reconstruction error magnitudes between the input variables and the class information variables.

The backpropagation (BP) algorithm updates the parameter set with Equation (17) until an optimal set is found.

### 4.2. Stacked DAE

To learn deep and more complex features, a single DAE may not be sufficient. Thus, an SDAE is constructed by hierarchically stacking several DAEs in a layer-by-layer manner. It is trained by two main procedures: pretraining and fine tuning.

For the pretraining procedure, the following steps are undertaken:

• For the first DAE, the raw training data ${\left\{\left(x_{i},y_{i}\right)\right\}}_{i=1}^{N}$, including the raw input data and the raw class information data, are exploited to pretrain the model. After this, $\theta^{1}=\left\{W^{1},b^{1},{\tilde{W}}_{x}^{1},{\tilde{b}}_{x}^{1},{\tilde{W}}_{y}^{1},{\tilde{b}}_{y}^{1}\right\}$, the parameter set in the first DAE, is learned, while the defect-related features in the first DAE $h^{1}$ are extracted in the hidden layer.
• For the second DAE, the extracted features from the first DAE and the raw class information data ${\left\{\left(h_{i}^{1},y_{i}\right)\right\}}_{i=1}^{N}$ are exploited to pretrain the model with a modified Equation (17),
  (18) $$J_{DAE2}\left(\theta^{2}\right)=\frac{1}{2N}\sum_{i=1}^{N}\left({∥h_{i}^{1}-{\tilde{h}}_{i}^{1}∥}^{2}+\lambda {∥y_{i}-{\tilde{y}}_{i}^{2}∥}^{2}\right)$$
  where ${\tilde{y}}^{2}=g\left({\tilde{W}}_{y}^{2}h^{1}+{\tilde{b}}_{y}^{2}\right)$. After this, $\theta^{2}=\left\{W^{2},b^{2},{\tilde{W}}_{x}^{2},{\tilde{b}}_{x}^{2},{\tilde{W}}_{y}^{2},{\tilde{b}}_{y}^{2}\right\}$, the parameter set in the second DAE, is learned, while the defect-related features in the second DAE, $h^{2}$, are extracted in the hidden layer.
• In turn, assume that the *k*th DAE has already been pretrained and $h^{k}$ as well as the set $\theta^{k}=\left\{W^{k},b^{k},{\tilde{W}}_{x}^{k},{\tilde{b}}_{x}^{k},{\tilde{W}}_{y}^{k},{\tilde{b}}_{y}^{k}\right\}$ has been obtained, then the (k+1)th DAE would be pretrained with the loss function
  (19) $$J_{DAE(k+1)}\left(\theta^{k+1}\right)=\frac{1}{2N}\sum_{i=1}^{N}\left({∥h_{i}^{k}-{\tilde{h}}_{i}^{k}∥}^{2}+\lambda {∥y_{i}-{\tilde{y}}_{i}^{k+1}∥}^{2}\right)$$
  where ${\tilde{y}}^{k+1}=g\left({\tilde{W}}_{y}^{k+1}h^{k}+{\tilde{b}}_{y}^{k+1}\right)$. The parameter set in the (k+1)th DAE $\theta^{k+1}=\left\{W^{k+1},b^{k+1},{\tilde{W}}_{x}^{k+1},{\tilde{b}}_{x}^{k+1},{\tilde{W}}_{y}^{k+1},{\tilde{b}}_{y}^{k+1}\right\}$, is then learned, while the high level defect-related features in the (k+1)th DAE $h^{k+1}$ are extracted in the hidden layer.

**Figure 5:**
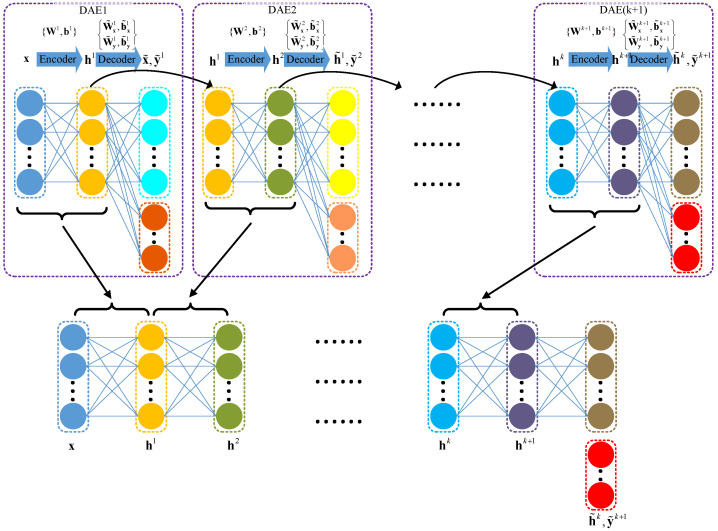
Schematic diagram of the pretraining procedure of layer-by-layer deep DAEs.

**Figure 6:**
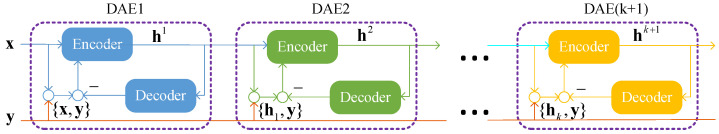
Flowchart of the pretraining procedure of the SDAE.

It is noted that, in the pretraining procedure, the class information variables are not included in the input for DAEs in each layer, although the reconstruction errors are based on the raw class information variables. The raw class information variables are treated as set values for increasingly deeper layers to extract more complex defect-related features. For a better understanding, Figure 5 illustrates a schematic diagram of the pretraining procedures of layer-by-layer deep DAEs, where the network below DAEs denote the defect-related features would be gradually reinforced with the increase in network layers. Additionally, Figure 6 presents a flowchart of the pretraining procedure of the SDAE.

To predict real-time defects, a back propagation neural network (BPNN) is constructed as a forecasting layer based on the deepest features learned by the SDAE (the top hidden layer). At this point, fine tuning of the whole network would be finally adopted to modify the network parameters to further improve the forecasting performance. Assume the hidden variables of BPNN $h^{BPNN}$ are nonlinearly mapped as

(20) $$h^{BPNN}=f^{BPNN}\left(W^{BPNN}h^{K}+b^{BPNN}\right)$$

where $f^{BPNN}$, $W^{BPNN}$, and $b^{BPNN}$ are the activation function, the weight parameter, and the bias parameter, respectively, from the input layer to the hidden layer. *K* denotes the total number of DAEs. Then, the forecasting result is calculated as

(21) $${\tilde{y}}^{BPNN}=g^{BPNN}\left({\tilde{W}}^{BPNN}h^{BPNN}+{\tilde{b}}^{BPNN}\right)$$

where $g^{BPNN}$, ${\tilde{W}}^{BPNN}$, and ${\tilde{b}}^{BPNN}$ are the activation function, the weight parameter, and the bias parameter from the hidden layer to the output layer.

### 4.3. SDAE-Based Forecasting

The SDAE-based forecasting network is finally built after pretraining and fine-tuning procedures. To make a prediction for a query sample $x_{new}$, the following features are successively learned,

(22) $$\begin{array}{cc} h_{new}^{1} & =f(W^{1}x_{new}+b^{1})\\ h_{new}^{2} & =f(W^{2}h_{new}^{1}+b^{2})\\ \cdots & \\ h_{new}^{k+1} & =f(W^{k+1}h_{new}^{k}+b^{k+1}),\;k=1,\cdots ,K-1 \end{array}$$

Then, the forecasting result, $y_{new}$, is obtained,

(23) $$\begin{array}{cc} h_{new}^{BPNN} & =f^{BPNN}\left(W^{BPNN}h_{new}^{K}+b^{BPNN}\right)\\ y_{new} & ={\tilde{y}}_{new}^{BPNN}=g^{BPNN}\left({\tilde{W}}^{BPNN}h^{BPNN}+{\tilde{b}}^{BPNN}\right) \end{array}$$

**Figure 7:**
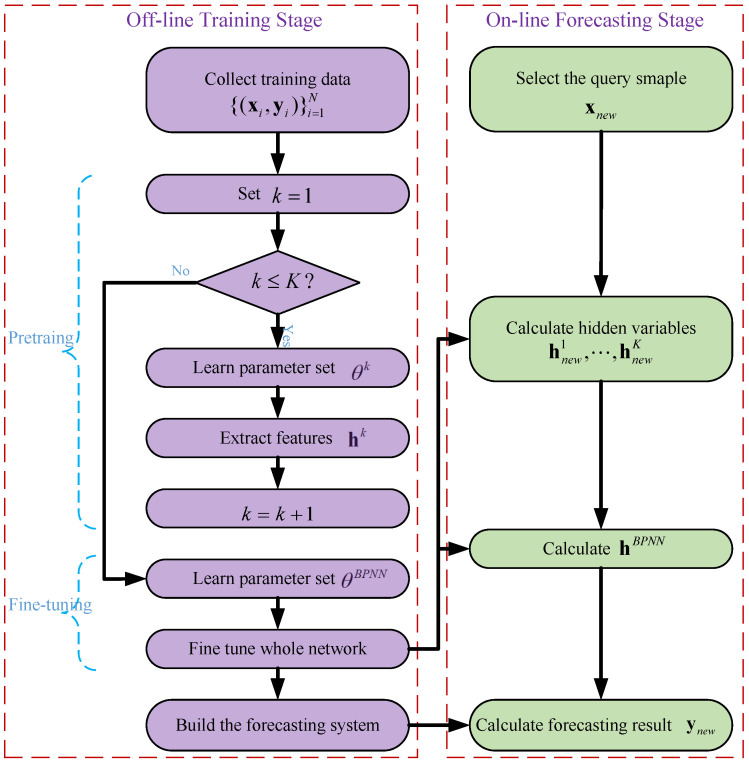
Flowchart of SDAE-based defect forecasting.

The detailed procedures of the offline training stage and online forecasting stage of the proposed SDAE-based forecasting method are listed in Algorithms 3 and 4, respectively, where $h^{0}=x$. To summarize, Figure 7 illustrates a flowchart of SDAE-based forecasting.
**Algorithm 3** Off-line Training Stage of the SDAE.1:**Pretraining procedure**:2:     Collect training data ${\left\{\left(x_{i},y_{i}\right)\right\}}_{i=1}^{N}$;3:     Set k=1;4:     **while** k≤K **do**5:          Construct k−th DAE structure with input variables $h^{k-1}$;6:          Initialize $\theta^{k}=\left\{W^{k},b^{k},{\tilde{W}}_{x}^{k},{\tilde{b}}_{x}^{k},{\tilde{W}}_{y}^{k},{\tilde{b}}_{y}^{k}\right\}$ randomly;7:          Learn parameter set $\theta^{k}$ using Equations (15), (16) and (19);8:          Extract features in hidden layer set $h^{k}$;9:          k=k+1;10:**Fine-tuning procedure**:11:     Construct BPNN network with input variables $h^{K}$;12:     Initialize $\theta^{BPNN}=\left\{W^{BPNN},b^{BPNN},{\tilde{W}}_{BPNN},{\tilde{b}}_{BPNN}\right\}$ randomly;13:     Learn parameter set $\theta^{BPNN}$ using Equations (19)–(21);14:     Fine tune the whole deep network with iterative propagations.15:     Construct the forecasting system using Equations (22) and (23).

**Algorithm 4** On-line Forecasting Stage of the SDAE.
1:Select the query sample $x_{new}$;2:Encode to obtain hidden variables $h_{new}^{1},\cdots ,h_{new}^{K}$ layer-by-layer using Equation (22);3:Calculate $h_{new}^{BPNN}$ using Equation (23);4:Calculate the corresponding forecasting result using (23).


The SDAE in this paper is naturally suitable for multi-class forecasting tasks. It should be mentioned that at the present stage, the predictor was expected to only binarily forecast whether the slab is defective or not, so there should only be one class information variable *y* ($R^{d_{y}}=1$), while the vector $y\in R^{d_{y}}$ degenerates to the scalar $y\in R^{1}$.

## 5. Case Studies and Comparisons

In this section, case studies are provided to demonstrate the feasibility and efficiency of the data-driven defect forecasting methods based on a real-life continuous casting process with eight types of casting slabs. The case studies also help to improve the understanding of the continuous casting process and the data characteristics. Both classical methods and the developed methods (SR-KDLS and SDAE-BPNN) are employed.

All the data were collected from the daily process records of a real-life continuous casting process in China. The subsurface inclusion defects were reported and recorded in subsequent production procedures. A total of 33 process variables were selected from all the available variables to construct the data-driven forecasting models according to the engineering experience, and are tabulated together in Table 1. Specifically, eight types of casting slab are included. One dataset was collected for each category with both normal data and defective data, as shown in Table 2. The sampling time ranges from 1 October 2018 through to 11 November 2018. It is noted that the numbers of samples vary a lot between different datasets, while the percentages of positive samples also vary a lot. Each dataset was randomly segmented into a training sub-dataset and a testing sub-dataset, roughly preserving similar percentages of positive and negative samples in both the training set and the testing set. The ratio of the number of training samples to the number of testing samples is approximate 7:3. Detailed configurations of the eight datasets are listed in Table 2, where the “Imbalance degree” denotes the ratio of the percentage of negative samples to the percentage of positive samples. The categories are ordered according to the imbalance degree from large to small. For proprietary reasons, other specific details about the process will not be further disclosed. A general descriptions of the continuous casting process can be found in Section 2.1.

It should also be noted that the percentage of negative samples is much higher than the percentage of positive samples for most original datasets (Table 2). Thus, this is an imbalanced forecasting problem, in which the model is very much inclined to the majority class [39,40,41]. As a result, the minority forecasting accuracy will be quite poor. Weiss pointed out that, in this case, the forecasting performance is much poorer than the general situation and the minority samples are easily treated as noise during training [42,43]. The SMOTE (synthetic minority oversampling technique), proposed by Wallace [44], increases the minority class by synthesizing new samples from the existing samples, not by simply oversampling the minority class. In this paper, SMOTE is employed to eliminate the imbalance problem. As a result, the ratio of the percentage of negative samples to the percentage of positive samples in the augmented training data should be approximately 1. All the models will be trained on the augmented training sub-dataset.

The hardware configuration is listed as follows: CPU: Intel(R) Core(TM) i9-12900K (16 cores); RAM: 32 GB × 2; no discrete graphics card. The software configuration is listed as follows: OS: Windows 10 (64 bit); Python 3.10.10; MATLAB(R) R2022a. For convenience and standardization, the numerical tools provided by “scikit-learn” (version 1.2.2), “xgboost” (version 1.7.4), and “imbalanced-learn” (version 0.10.1) [23,45,46] are utilized, such as “train_test_split” and “GridSearchCV” in “sklearn.model_selection”; “SMOTE” in “imblearn”; “LinearDiscriminantAnalysis”, “QuadraticDiscriminantAnalysis”, and “LogisticRegression” in “sklearn.linear_model”; and “XGBClassifier” in “xgboost”.

### 5.1. Parameter Selection

To obtain the highest forecasting performance possible, the parameter optimization method was designed to choose a set of optimal parameters for a learning algorithm. There are no parameters to be tuned when using LDA and LR.

For SVM, the radial basis function (Gaussian) kernel $\kappa \left(x_{i},x_{j}\right)=exp\left(-\frac{\left|\right|x_{i}-x_{j}{\left|\right|}^{2}}{2\delta^{2}}\right)$ is used as it is robust to parameter variations and has infinite degrees of freedom. To determine the optimal value of kernel width, a rough value is chosen by $\upsilon =1/2\delta^{2}=c\times m\times \sigma^{2}$ according to [47], where *m* and σ are the dimension of the input space and the variance of training data, respectively. Then, the final value would be exhaustively adjusted around the rough value. To exploit the training data as much as possible, k-fold cross-validation is recommended instead of splitting an independent validation dataset.

For XGBoost, this paper introduces an intuitionistic and highly efficient method based on a grid search [48] to determine the parameters of maximum depth, minimum sum of instance weight, maximum delta step, subsample, and learning rate. It is noted that the evaluating metrics should be chosen according to the practical situation and requirements, such as precision, recall, area under curve (AUC), and other model evaluation metrics. Specifically, the maximum depth and minimum sum of instance weight are searched among “1, 2, 3, 4, 5, 6” and “1, 2, 3, 4”, respectively, while other parameters are fixed (by default, learning rate = 0.2, subsample = 1, and maximum delta step = 0.7). After that, the subsample and maximum delta step are searched among “0.6, 0.7, 0.8, 0.9, 1.0” and “0, 1, 2, 3, 4”, respectively, with the tuned maximum depth and minimum sum of instance weight and learning rate = 0.2. Finally, the learning rate is searched among “0.05, 0.10, 0.15, 0.20, 0.25, 0.30, 0.40, 0.50”.

For SDAE, considering that the dimensions of the input variables and class information variables are 33 and 1, respectively, three DAEs are stacked to construct a seven-layer deep network, while the numbers of the hidden variables are set to 30, 25, and 20. For BPNN, one hidden layer is set with 15 hidden variables. The dimensions of the input variables of BPNN are equal to the number of the deepest hidden variables of SDAE, i.e., 20. The dimensions of the output variables of BPNN are equal to the dimensions of the class information variable, i.e., 1. The reciprocal of λ is equal to the imbalance degree plus 1. In this paper, the basic BPNN, SAE-BPNN, and SDAE-BPNN are considered. It should be noted that the activation function provides a curvilinear match between the input and output layers and also determines the output of the cell by processing the net input to the cell [49,50,51,52,53]. In this paper, the widely used sigmoid function is selected as the activation function for artificial neural networks for primitive comparisons and verification, which may not give optimal results without any validation to choose the activation function for specific data. The learning rate is set to 0.01, which empirically works well in all the repeated simulations in this work. The locks are removed when updating operations in the optimizer “tf.train.GradientDescentOptimizer”.

For SR-KDLS, the forms of the kernel function and kernel parameter are set to be the same as SVM for a fair comparison. γ is chosen by the grid search from the candidate set with an exponential sequence $\left\{5\times 10^{e}|e=-8,-7,\ldots ,7,8\right\}$.

### 5.2. Results and Analysis

The forecasting results of all eight data-driven forecasting methods are tabulated in Table 3, where the highest and second-highest F1 scores for each category are in bold and underlined, respectively. It is emphasized that all the data-driven forecasting methods are trained with identical samples for each category of slabs to eliminate randomness; technically, the SMOTE uses the same random state.

#### 5.2.1. Overall Analysis

Overall, it can be easily found that the forecasting results in Table 3 provide preliminary verification and demonstration of the feasibility and efficiency of the data-driven methods. Unlike traditional methods based on a mechanism model, physical parameters, or process knowledge (metallurgy function), data-driven methods do not need precise mechanistic information or expert knowledge; they merely rely on abundant process data to provide a precise, straightforward, economical, and universal forecasting performance.

However, the performances of different data-driven methods vary a lot. Based on F1 metrics, the developed SR-KDLS and SDAE-BPNN intuitively perform better than other methods. LDA is conducted upon the restrictive assumptions of multivariate normal distribution and linearity, which are very likely contrary to the practical situation. The use of LR removes the multivariate normal distribution assumption; however, it may suffer from under-fitting. SVM is one of the most classical machine learning algorithms and is characterized by nonlinear mapping, a maximum forecasting gap, and robustness. However, it only focuses on the forecasting boundary and does not consider the within-class scatter. XGBoost is as a powerful decision-tree-based ensemble machine learning algorithm. The results in Table 3 verify the efficiency of XGBoost. However, its flaw is that it contains too many parameters and thus cannot be artificially tuned. Although this paper introduces an intuitionistic method based on practical evaluation metrics to determine the parameters for XGBoost, it is still an open question to obtain more adapted parameters.

The developed SDAE, a deep learning method, is derived from AEs and SAEs. However, AEs are a shallow network which cannot extract complex features. SAEs cannot guarantee the extraction of defect-related features, since they ignore defect-related information. In the SDAE, each DAE is designed by minimizing the reconstruction error of both the input variables and the class information variables, such that defect-related features are guaranteed to be extracted. All the DAEs are hierarchically stacked to learn increasingly deeper defect-related features which can greatly improve the forecasting performance. When combined with a BPNN, SDAE-BPNN more accurately forecasts subsurface inclusion defects. Comparing the results between BPNN, SAE-BPNN, and SDAE-BPNN in Table 3, BPNN clearly performs the worst as it has a shallow structure. By considering defect-related information, the SDAE-BPNN captures more valuable representations than SAE-BPNN. The results confirm that SDAE-BPNN performs better than SAE-BPNN.

Traditional data-driven forecasting methods mainly focus on discovering low-dimensional embeddings with a certain class separability, and the forecasting result is calculated by supplementary classifiers based on these embeddings. In this paper, the SR-KDLS is designed as a more suitable model for forecasting as it directly provides forecasting information, instead of low-dimensional embeddings. It exploits the discriminative information in the scatter-regularization term of optimization to pursue both intra-class compactness and inter-class separability. It has the ability to deal with nonlinear data by integration of the kernel function, and with the help of the Representer Theorem for RKHS, an analytical solution can be pursued without iterative procedures. The forecasting results verify and demonstrate its feasibility and efficiency.

#### 5.2.2. Discussion of Imbalance Degree

**Figure 8:**
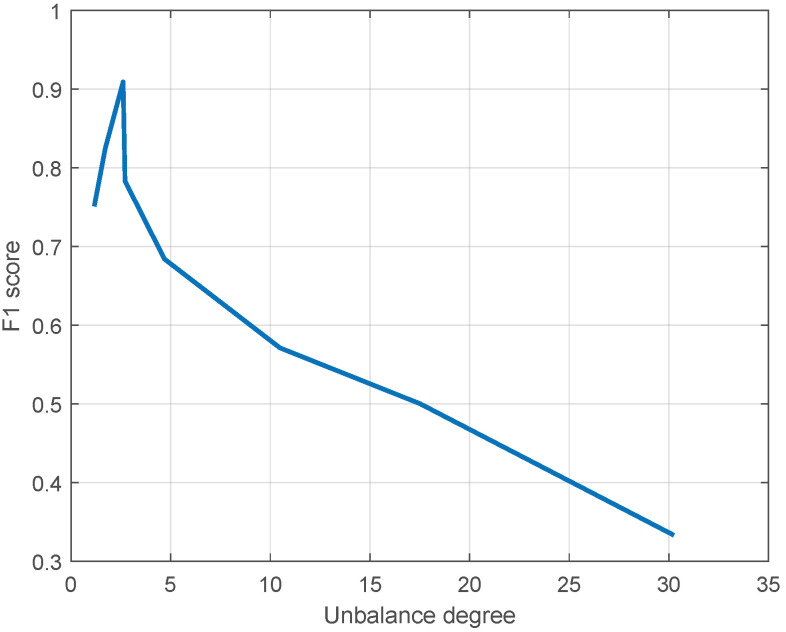
The F1 score of SR-KDLS versus the imbalance degree.

It is noted that the F1 scores of data-driven forecasting methods are inversely related (approximately) to the imbalance degree of the dataset, especially when the imbalance degree is large. Figure 8 shows the F1 score of SR-KDLS (on behalf of the involved data-driven methods) versus the imbalance degree. Qualitatively, when the imbalance degree is large, the information of the minority class is too scarce to train a precision model. As the imbalance degree decreases towards 1, the imbalanced problem becomes less severe (if the imbalance degree is equal to 1, the imbalance problem disappears); thus, more information on the minority class is available. The relation is not strictly negative when the imbalance degree is small (still greater than 1), since the imbalance problem is no longer the decisive factor in this situation.

Table 3 shows that when the imbalance degree is large, the forecasting performance is not very satisfactory. It is known that sufficient information is necessary for valid forecasting results. As the information in the minority class is insufficient, the SMOTE is employed. The SMOTE works by selecting examples that are close in the feature space, drawing a line between the examples in the feature space, and randomly drawing a new sample at a point along the line to increase the minority class. Without the SMOTE, all models would be severely biased towards the majority class (the results without SMOTE are omitted, since they are obviously worse). Although some new information is introduced and the performances of the data-driven models are greatly improved, the randomly augmented information is unreliable and may sometimes be inconsistent with the practical process. New data-augmentation techniques for specific processes are expected to further improve the forecasting performance when there is a large imbalance degree.

#### 5.2.3. Calculation Time

As this paper focuses on real-time forecasting, the timing of the models is worth an inspection. Table 4 details the calculation time of the whole modeling procedure, marked as “Offline”, as well as the mean calculation time of forecasting per one testing sample, marked as “Online”. The training durations for all models are acceptable in real applications; the longest is less than 2 min (SDAE-BPNN for Category 5). All the online forecasting times are less than 0.1 ms per sample, which is much faster than the shortest sampling period of commonly used process variables in the continuous casting process. It is shown that all models meet the time requirements of real-time forecasting. For traditional detectors, defects are detected when the slabs have already been rolled by subsequent hot rolling or cold rolling process, leading to a large time delay between defect generation and defect detection. With the help of data-driven modeling and prediction methods, the defects can be predicted in real time during the manufacture of continuous casting slabs. Note that it takes a lot longer to train the developed SR-KDLS and much longer to train the developed SDAE-BPNN than traditional methods, especially with Category 5. However, this computational complexity is far from being unacceptable since the training is conducted offline; it is not related to online forecasting procedures. It is worth having high-accuracy SDAE-BPNN and SR-KDLS models at the cost of some offline computational complexity.

All the results of the comparison case studies have verified and demonstrated the feasibility and efficiency of the five representative data-driven methods and also the improvement demonstrated by the developed SR-KDLS and SDAE-BPNN in forecasting subsurface inclusion defects. Table 3 in Section 5.2.1 shows that the defects are accurately predicted, while Table 4 in Section 5.2.3 shows that the defects are timely predicted.

## 6. Outlook

Real-time forecasting of subsurface inclusion defects, from an industrial perspective, needs further research. Some outlooks to further improve the subsurface inclusion defect forecasting performance are given in the following.

• Feature engineering. There is a saying that is widely circulated in the industry: data and features determine the upper limit of machine learning, and models and algorithms approach this upper limit. Feature engineering is fundamental to the application of machine learning; this can be carried out either manually upon domain knowledge or automatically, which is called automated feature learning. This paper primitively uses all the original process variables (features) to train the data-driven model. A better forecasting performance would be obtained with elaborate features. It would be helpful to extract feature characteristics or to reduce the dimensionality with a manifold learning algorithm.
• Activation function. The selection of an appropriate activation function significantly affects artificial neural network performance. There are many types of activation function, such as the threshold function, step activation function, sigmoid function, and hyperbolic tangent function. This paper simply sets the activation function as a sigmoid function. However, future work should include an analysis of different activation functions, and the function that gives the best performance should be utilized. One method could be to evaluate the multivariate distribution of the input variables by performing a goodness-of-fit test.
• Time delay estimation. As subsurface inclusion defects cannot be detected by traditional detectors until the slabs have already been rolled in subsequent hot rolling or cold rolling processes, there exists a large time delay between process variable measurements and defective information gathering; thus, alignment of data is necessary. In this paper, data are aligned according to a rough estimate of the time delay by operation experiences and logs; thus, the data may not be accurately aligned, which presents an obstacle in the construction of a data-based model with high precision. A variable time delay estimation technique is worth studying in this situation.
• Imbalanced data. The imbalance problem is obvious when training forecasting models, since there are usually many more normal (negative) samples than defective (positive) samples. Although some tools (such as down-sampling, SMOTE, and cost-sensitive learning) are designed to handle this problem, they are flawed by the failure to generate reliable new information in the data domain and only simply combine the original information or randomly generate information. New data-augmentation techniques for specific processes to increase reliable information are expected to further improve the forecasting performance in the case of a large imbalance degree. Generative adversarial networks (GANs) may be a promising solution. In addition, synthesizing data by transferring information from data-intensive regions to data-scarce regions may also help to enhance the forecasting performance.
• Data-driven methods fused by process knowledge. Although data-driven methods do not need precise mechanistic information or expert knowledge, available process knowledge, which may not or may only partially be inferred in the collected data, would further help to design a targeted model with a greatly improved forecasting performance. Additionally, process knowledge would also help to increase the minority class with reliable information, as well as estimate an accurate time delay.

## 7. Conclusions

Real-time forecasting of subsurface inclusion defects for continuous slab casting is of great significance to the steel industry. It is, however, a hard task. This paper introduces data-driven methods to solve this problem and presents a comparative study. In order to design more suitable models and improve the subsurface inclusion defect forecasting performance, the SR-KDLS model and SDAE-BPNN model were developed. The former is a kernel discriminant analysis method, and the latter is a deep neural network method. Case studies were carried out based on a real-life continuous casting process where the imbalance degree drastically ranged from 1 to 30 in different categories. The feasibility and efficiency of the data-driven methods are demonstrated; the defects could be predicted within 1 ms with acceptable F1 scores. For example, XGBoost achieves a 71.1% F1 score when the imbalance problem is mild (category 8, imbalance degree 1.16). Moreover, experiments show that the forecasting performance is further improved in the developed SR-KDLS and SDAE-BPNN methods without much computational burden; the F1 scores are obviously higher than those for the common data-driven methods. For example, the F1 scores are 91.2% and 90.9% for SR-KDLS and SDAE-BPNN, respectively, for category 6, while SVM only achieves a 71.4% F1 score. All the prediction procedures for the developed SR-KDLS and SDAE-BPNN methods take less than 0.1 ms per sample; there will be hardly any delay in real-life application of the models considering the manufacturing procedure of continuous slab casting.

A real industry issue—real-time forecasting of subsurface inclusion defects—is the focus of this study and has been discussed in detail. This study not only improves the performance of subsurface inclusion defect forecasting, but also offers potential economic benefits for the steel industry.

## Figures and Tables

**Table 1:** Process variables of the real-life continuous casting process.

No.	Description	Type	No.	Description	Type
V01	Ladle weight	float	V18	Mold water flow (east)	float
V02	Ladle temperature	float	V19	Mold input water pressure	float
V03	Ladle shroud Ar flow	float	V20	Mold out water pressure (north)	float
V04	Ladle shroud Ar pressure	float	V21	Mold out water pressure (south)	float
V05	Tundish weight	float	V22	Mold out water pressure (west)	float
V06	Tundish temperature	float	V23	Mold out water pressure (east)	float
V07	Tundish Ar flow 1	float	V24	Mold actual open (north)	float
V08	Tundish Ar flow 2	float	V25	Mold actual open (south)	float
V09	Tundish Ar pressure	float	V26	Mold actual open (west)	float
V10	Mold input water temperature	float	V27	Mold actual open (east)	float
V11	Mold output water temperature (north)	float	V28	Speed (set)	integer
V12	Mold output water temperature (south)	float	V29	Speed (actual)	integer
V13	Mold output water temperature (west)	float	V30	Up nozzle Ar flow (5L)	float
V14	Mold output water temperature (east)	float	V31	Up nozzle Ar pressure	float
V15	Mold water flow (north)	float	V32	Stopper Ar flow	float
V16	Mold water flow (south)	float	V33	Stopper Ar back pressure	float
V17	Mold water flow (west)	float			

**Table 2:** Configurations of the eight datasets: (a) original dataset, and (b) training sub-dataset and testing sub-dataset.

	(a)							
**No.**	**Original Dataset**							
	**Numbers of Samples**		**Percentage of Negative Samples**		**Percentage of Positive Samples**		**Imbalance Degree**	
1	1198		96.8%		3.2%		30.250	
2	148		94.6%		5.4%		17.519	
3	1538		91.2%		8.7%		10.483	
4	262		82.4%		17.6%		4.682	
5	6058		73.0%		27.0%		2.704	
6	112		72.3%		27.7%		2.61	
7	239		63.2%		36.8%		1.717	
8	422		53.8%		46.2%		1.16	
	**(b)**							
**No.**	**Training Sub-Dataset**				**Testing Sub-Dataset**			
	**Numbers of Samples**	**Perc. of Negative Samples**	**Perc. of Positive Samples**	**Imbalance Degree**	**Numbers of Samples**	**Perc. of Negative Samples**	**Perc. of Positive Samples**	**Imbalance Degree**
1	838	96.5%	3.5%	27.57	360	97.5%	2.5%	39
2	103	94.2%	5.8%	16.24	45	95.6%	4.4%	21.73
3	1076	91.4%	8.6%	10.63	462	90.9%	9.1%	9.90
4	183	83.6%	16.4%	5.10	79	79.7%	20.3%	3.93
5	4240	73.0%	27.0%	2.70	1818	73.0%	27.0%	2.70
6	78	74.4%	25.6%	2.91	34	67.6%	32.4%	2.09
7	167	61.7%	38.3%	1.61	72	66.7%	33.3%	2.00
8	295	57.3%	42.7%	1.34	127	45.7%	54.3%	0.84

**Table 3:** Forecasting results of the data-driven forecasting methods.

No	LDA			LR			SVM			XGBoost		
	**Prec.**	**Recall**	**F1**	**Prec.**	**Recall**	**F**1	**Prec.**	**Recall**	**F1**	**Prec.**	**Recall**	**F1**
1	4.9%	55.5%	9.0%	2.8%	44.4%	5.3%	3.1%	44.4%	5.9%	22.2%	22.2%	22.2%
2	18.2%	100%	30.8%	11.8%	100%	21.1%	2.6%	50.0%	4.9%	50.0%	50.0%	**50.0%**
3	18.4%	64.3%	28.6%	14.9%	69.0%	24.5%	11.4%	57.1%	19.0%	52.5%	50.0%	51.2%
4	48.0%	75.0%	58.5%	50.0%	75.0%	60.0%	46.9%	93.8%	62.5%	54.5%	37.5%	44.4%
5	60.4%	75.8%	67.2%	48.6%	70.5%	57.5%	42.2%	76.2%	54.3%	65.0%	70.1%	67.5%
6	56.3%	81.8%	66.7%	50.0%	72.7%	59.3%	58.8%	90.9%	71.4%	100%	81.8%	90.0%
7	61.3%	79.2%	69.1%	71.4%	83.3%	76.9%	33.9%	83.3%	48.2%	72.7%	66.7%	69.6%
8	75.0%	73.9%	74.5%	70.9%	81.2%	75.7%	63.0%	91.3%	74.6%	72.7%	69.6%	71.1%
**No**	**BPNN**			**SAE-BPNN**			**SDAE-BPNN**			**SR-KDLS**		
	**Prec.**	**Recall**	**F1**	**Prec.**	**Recall**	**F1**	**Prec.**	**Recall**	**F1**	**Prec.**	**Recall**	**F1**
1	3.6%	100%	6.9%	22.2%	22.2%	22.2%	40.0%	22.2%	28.6%	66.7%	22.2%	**33.3%**
2	4.5%	100%	8.7%	16.7%	100%	28.6%	22.2%	100%	36.4%	50.0%	50.0%	**50.0%**
3	9.4%	97.6%	17.1%	64.3%	42.9%	51.4%	60.5%	54.8%	**57.5%**	71.4%	47.6%	57.1%
4	40.0%	37.5%	38.7%	44.4%	50.0%	47.1%	45.8%	68.8%	55.0%	59.1%	81.3%	**68.4%**
5	29.5%	88.4%	44.2%	72.2%	62.9%	67.2%	74.2%	65.0%	69.2%	75.0%	81.8%	**78.3%**
6	45.0%	81.8%	58.1%	68.8%	100%	81.5%	84.6%	100%	**91.2%**	90.9%	90.9%	90.9%
7	32.8%	91.7%	48.4%	70.0%	58.3%	63.6%	66.7%	75.0%	70.6%	75.0%	91.7%	**82.5%**
8	57.5%	72.5%	64.1%	78.0%	66.7%	71.9%	80.6%	78.3%	**79.4%**	62.5%	94.2%	75.1%

The highest and second-highest F1 scores for each category are in bold and underlined, respectively.

**Table 4:** Calculation time of different methods (milliseconds).

	Method	LDA		LR		SVM		XGBoost	
No.									
		**Offline**	**Online**	**Offline**	**Online**	**Offline**	**Online**	**Offline**	**Online**
1		3.987	<0.100	25.917	<0.100	53.348	<0.100	32.891	<0.100
2		1.993	<0.100	4.982	<0.100	0.992	<0.100	16.944	<0.100
3		3.986	<0.100	23.921	<0.100	79.735	<0.100	36.876	<0.100
4		0.997	<0.100	19.934	<0.100	1.994	<0.100	17.941	<0.100
5		11.960	<0.100	32.932	<0.100	806.321	<0.100	68.226	<0.100
6		0.997	<0.100	4.978	<0.100	0.997	<0.100	18.937	<0.100
7		1.996	<0.100	4.925	<0.100	1.994	<0.100	14.950	<0.100
8		2.006	<0.100	22.946	<0.100	2.990	<0.100	14.951	<0.100
	**Method**	**BPNN**		**SAE-BPNN**		**SDAE-BPNN**		**SR-KDLS**	
**No.**									
		**Offline**	**Online**	**Offline**	**Online**	**Offline**	**Online**	**Offline**	**Online**
1		1330.552	<0.100	2993.018	<0.100	23,163.590	<0.100	111.316	<0.100
2		28.904	<0.100	530.228	<0.100	3227.460	<0.100	51.690	<0.100
3		2204.629	<0.100	3589.460	<0.100	27,572.919	<0.100	164.479	<0.100
4		60.796	<0.100	569.234	<0.100	3061.574	<0.100	10.041	<0.100
5		5254.771	<0.100	13,523.745	<0.100	11,1557.549	<0.100	3215.067	<0.100
6		21.926	<0.100	182.774	<0.100	301.108	<0.100	4.243	<0.100
7		382.720	<0.100	581.570	<0.100	3087.267	<0.100	2.932	<0.100
8		726.571	<0.100	963.562	<0.100	5771.627	<0.100	30.998	<0.100

## Data Availability

The data presented in this study are available upon request from the corresponding author.
